# Terminal Continuation (TC) RNA Amplification Enables Expression Profiling Using Minute RNA Input Obtained from Mouse Brain

**DOI:** 10.3390/ijms9112091

**Published:** 2008-10-31

**Authors:** Melissa J. Alldred, Shaoli Che, Stephen D. Ginsberg

**Affiliations:** 1 Center for Dementia Research, Nathan Kline Institute, New York University School of Medicine, 140 Old Orangeburg Road, Orangeburg, New York 10962, USA; 2 Department of Psychiatry and; 3 Department of Physiology & Neuroscience, New York University School of Medicine, Orangeburg, New York 10962, USA

**Keywords:** aRNA, brain extract, column filtration, in vitro transcription, microarray, TC RNA amplification

## Abstract

A novel methodology named terminal continuation (TC) RNA amplification has been developed to amplify RNA from minute amounts of starting material. Utility of the TC RNA amplification method is demonstrated with two new modifications including obviating the need for second strand synthesis, and purifying the amplification template using column filtration prior to in vitro transcription (IVT). Using four low concentrations of RNA extracted from mouse brain (1, 10, 25 and 50 ng), one round TC RNA amplification was compared to one round amplified antisense RNA (aRNA) in conjunction with column filtration and drop dialysis purification. The TC RNA amplification without second strand synthesis performed extremely well on custom-designed cDNA array platforms, and column filtration was found to provide higher positive detection of individual clones when hybridization signal intensity was subtracted from corresponding negative control hybridization signal levels. Results indicate that TC RNA amplification without second strand synthesis, in conjunction with column filtration, is an excellent method for RNA amplification from extremely small amounts of input RNA from mouse brain and postmortem human brain, and is compatible with microaspiration strategies and subsequent microarray analysis.

## 1. Introduction

The advent of high-throughput microarray analysis has enabled significant progress in expression profiling studies in many disciplines including cancer biology, development, and neuroscience, among others. A disadvantage of conventional microarray technology is a requirement for significant amounts of high quality input sources of RNA for sensitivity and reproducibility. The quantity of RNA harvested from a single cell, estimated to be approximately 0.1–1.0 picograms, is not enough to employ standard RNA extraction procedures [1, 2]. Therefore, molecular biological methods have been implemented to increase the amount of input RNA for downstream genetic analyses, including exponential PCR-based amplification and linear RNA amplification procedures. PCR-based protocols are not optimal, as exponential amplification can skew the original quantitative relationships between genes from an initial population [3, 4]. In contrast, linear RNA amplification methods allow for the analysis of relative gene expression levels. A linear RNA amplification procedure typically entails generating quantities of RNA species through *in vitro* transcription (IVT) [4–6], although PCR/linear RNA amplification hybrid methods [7, 8] as well as isothermal RNA amplification [9, 10] procedures are also available that generate a faithful representation of the original input RNA.

**Figure 1.**
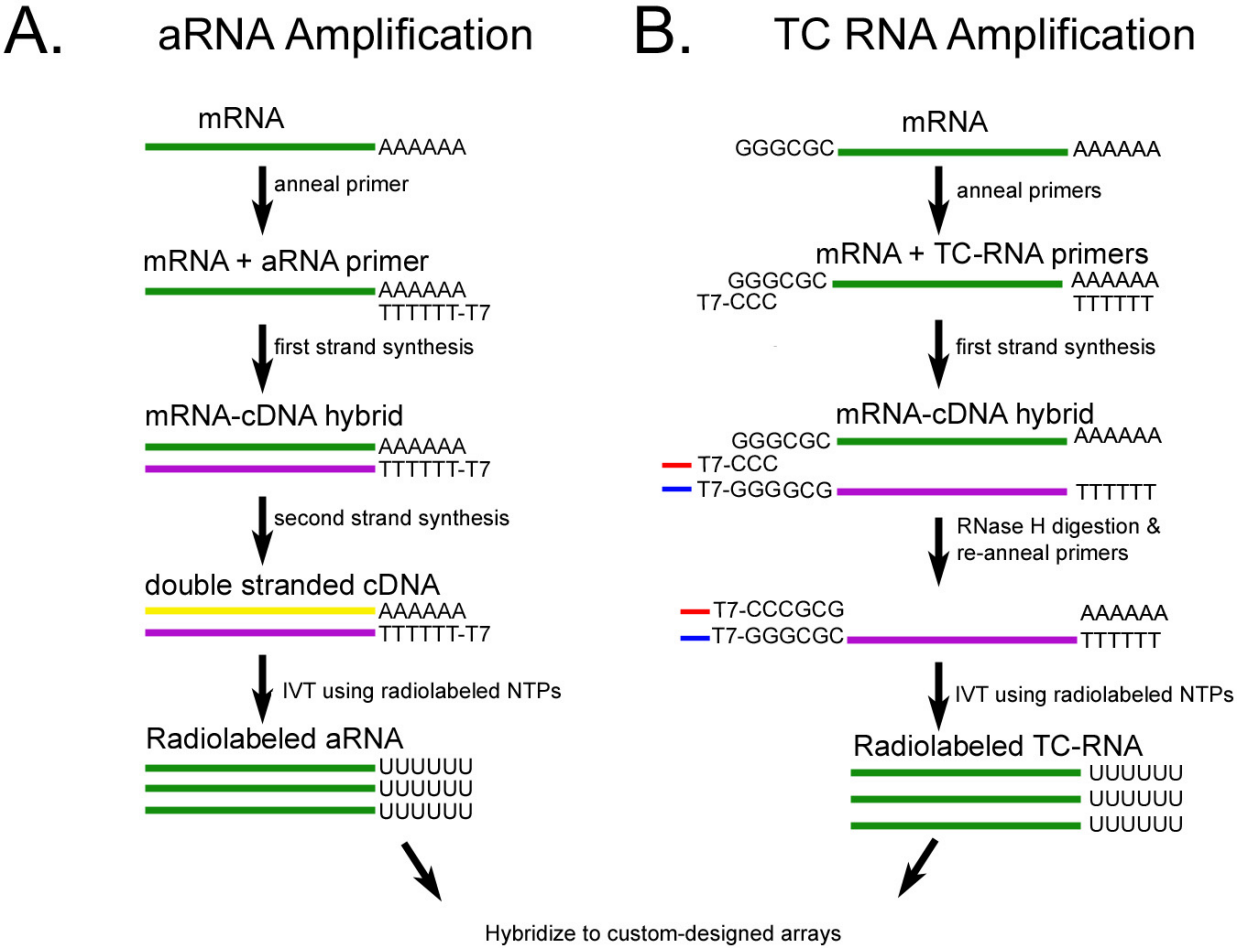
Schematic representation of the aRNA (left panel) and TC RNA amplification (right panel) methods as they are applied to custom-designed cDNA array analysis.

A well known linear amplification method, amplified antisense RNA (aRNA) amplification [2, 3, 5], enables the quantitation of relative gene expression levels from fairly minute amounts of input RNA (Figure 1). aRNA maintains a proportional representation of the size and complexity of input mRNAs. Each round of aRNA results in an approximate thousand fold amplification from the original amount of polyadenylated mRNA, although two rounds of amplification are typically required to generate sufficient quantities of aRNA for subsequent downstream analyses. Several aRNA-based amplification kits are available commercially.

A variety of strategies have been developed to improve RNA amplification efficiency [7, 8, 11–13]. An obstacle that hinders RNA amplification protocols is difficulty with second strand synthesis efficiency and specificity [4, 7, 8, 14]. Technical improvements include obviating second strand cDNA synthesis and enabling flexibility in placement of bacteriophage transcriptional promoter sequences for both antisense and sense amplification. The terminal continuation (TC) RNA amplification technology satisfies these objectives (Figure 1). TC RNA amplification of genetic signals originally included synthesizing first strand cDNA complementary to the mRNA template, subsequent second strand cDNA synthesis complementary to the first strand cDNA, and ensuing IVT using the synthesized double stranded cDNA template [6]. First strand cDNA synthesis complementary to the template mRNA requires two oligonucleotide primers, a first strand poly d(T) primer and a TC primer [6]. One round of amplification is sufficient for downstream genetic analyses. With the TC RNA amplification procedure, transcript orientation can be antisense (similar to conventional aRNA methods) when the bacteriophage promoter sequence is placed on the first strand poly d(T) primer or in a sense orientation when the promoter sequence is attached to the TC primer. Regional and single cell gene expression studies within the brains of animal models and human postmortem brain tissues have been performed via microarray analysis coupled with TC RNA amplification [15–19].

A modification of the TC RNA amplification procedure is presented that allows for robust RNA amplification without the need for second strand synthesis using mouse brain tissue [20]. Furthermore, cDNA purification strategies (drop dialysis and column purification) are compared and contrasted in the context of microarray performance of TC RNA amplification without second strand synthesis and conventional aRNA methodology, a technique used by our research group for many years [21, 22] prior to developing the TC RNA amplification strategy.

## 2. Results

Utilizing one round TC RNA amplification without second strand synthesis and aRNA amplification, signal intensity of 576 cDNAs corresponding to genes of interest for our research program was compared and contrasted on a custom-designed array platform with a gradient from 1 ng to 50 ng of input RNA from mouse brain. In addition, two different purification methods (column filtration and drop dialysis) were evaluated in conjunction with the aforementioned RNA amplification procedures.

**Figure 2.**
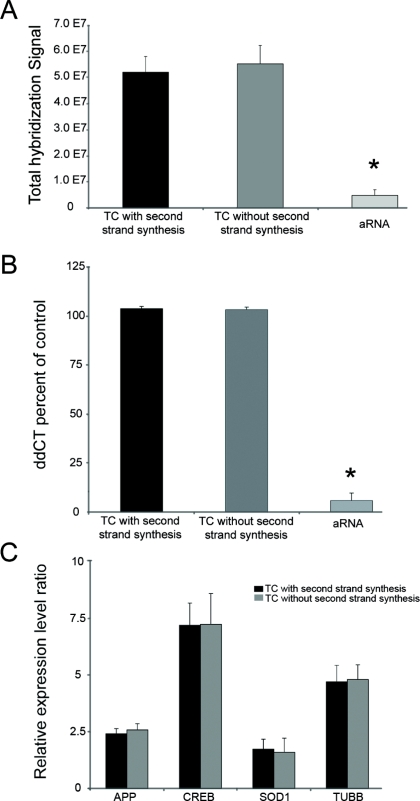
Comparison of the TC RNA amplification protocols and aRNA amplification using 50 ng of mouse brain RNA on specific gene expression levels. Key: APP, amyloid-beta precursor protein; CREB, cAMP response element binding protein; SOD1, superoxide dismutase; TUBB, beta tubulin.

A comparison of the RNA amplification methods indicates that the TC RNA amplification procedure without second strand synthesis generated robust hybridization signal intensity that did not differ significantly from the TC RNA amplification with second strand synthesis. A histogram (Figure 2A) indicates no significant differences between the original TC RNA amplification method with second strand synthesis and TC RNA amplification without second strand synthesis in terms of total hybridization signal intensity ± standard deviation. In contrast, both TC RNA amplification protocols display highly statistically significant increases in total hybridization signal intensity compared to aRNA amplification (asterisk denotes p < 0.0001). Depicted assays were performed with 50 ng of mouse brain RNA using the original TC RNA amplification protocol (n = 6), modified TC RNA amplification protocol (n = 7), and aRNA amplification (n = 5). Validation of the microarray observations was provided using qPCR for beta actin (ACTB; Figure 2B) and the AMPA glutamate receptor 1 (GRIA1; not shown). Specifically, a histogram depicts no significant differences in ACTB qPCR products between the two TC RNA amplification protocols (with second strand synthesis, n = 4; without second strand synthesis, n = 5) using 50 ng of input mouse brain RNA. Similar to the microarray findings, significantly less ACTB qPCR products are observed following aRNA amplification (n = 4; asterisk denotes p < 0.001; Figure 2B). Moreover, no significant differences in expression levels in representative genes with varying levels of hybridization signal intensity (Figure 2C) were observed in mouse brain. Similar results were garnered for individual clones using postmortem human brain as input RNA (data not shown).

**Figure 3.**
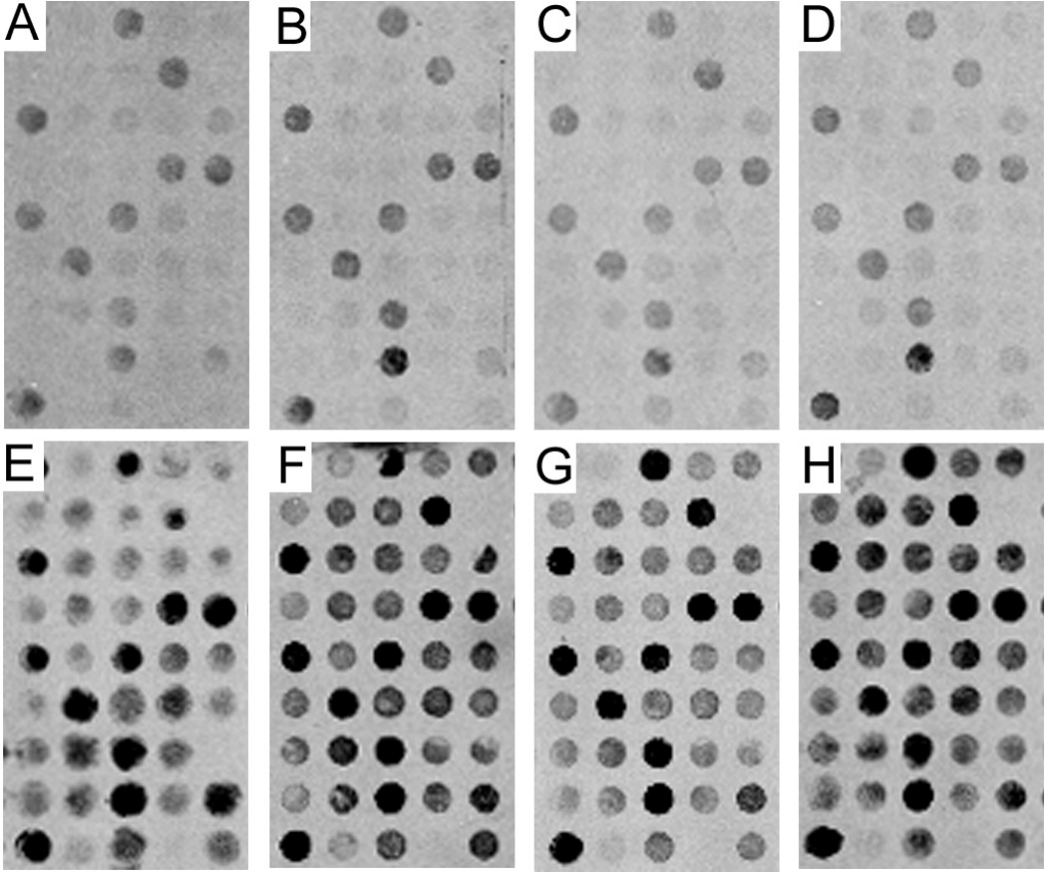
Custom-designed cDNA array analysis utilizing aRNA (A-D) and TC RNA amplification without second strand synthesis (E-H) in conjunction with drop dialysis purification.

In contrast, aRNA amplification on the identical mouse brain samples yielded a significantly lower total hybridization signal intensity result than either TC RNA amplification protocol (p < 0.0001). Moreover, the TC RNA amplification procedure produced an exponentially higher hybridization signal intensity for each individual cDNAs/ESTs examined when compared to the aRNA method starting from mouse brain RNA extracts of 1 ng, 10 ng, 25 ng, and 50 ng. Representative identical portions of membrane arrays are presented for aRNA with 1 ng (Figure 3A), 10 ng (Figure 3B), 25 ng (Figure 3C), and 50 ng (Figure 3D) of mouse brain input RNA. By comparison, the TC RNA amplification method using the same amount of mouse whole brain input RNA 1 ng (Figure 3E), 10 ng (Figure 3F), 25 ng (Figure 3G), and 50 ng (Figure 3H) displays a highly significant increase in hybridization signal intensity. All array phosphor scan images were adjusted to the same brightness and contrast levels for data acquisition and analysis.

**Figure 4.**
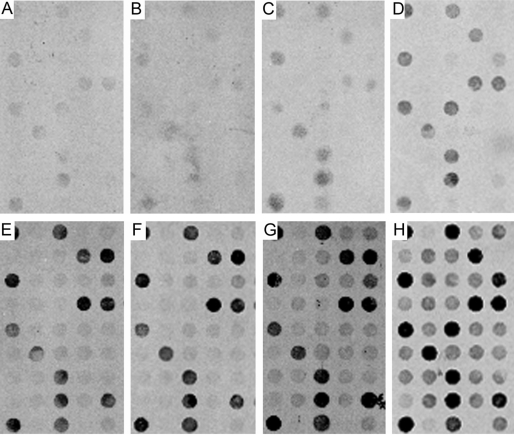
Comparison of the aRNA method (A-D) and the TC RNA amplification method (E-H) following column filtration purification using mouse brain RNA as an input tissue source.

Similar results were generated comparing aRNA with the TC RNA amplification method using the column purification method (Figure 4). Specifically, representative portions of custom-designed cDNA arrays depicting column filtration purification in conjunction with the aRNA method (Figure 4A-D) are presented in stark contrast to the results obtained using the TC RNA amplification method (Figure 4E-H) on parallel sets of samples. Note the low hybridization signal intensity for individual cDNAs/ESTs using the aRNA method as compared to the robust hybridization signal intensity obtained utilizing the TC RNA amplification without second strand synthesis protocol (Figure 4).

**Figure 5.**
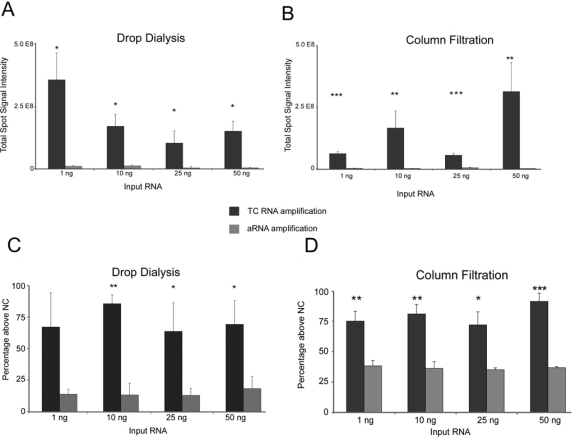
TC RNA amplification and aRNA amplification following drop dialysis (A, C) and column filtration (B, D) as a function of hybridization signal intensity (A, B) and percentage above negative control (NC) values (C, D). Single asterisk denotes (p < 0.05), double asterisk denotes (p < 0.01), and triple asterisk denotes (p < 0.001).

A statistically significant increase in total hybridization signal intensity for the TC RNA amplification method without second strand synthesis was observed as compared to the aRNA method at all concentrations studied, regardless of the purification method (Figure 5). Notably, the aRNA method displays significantly lower hybridization signal intensity above negative control signal values than the TC RNA amplification procedure at all four concentrations for the two purification procedures. Essentially, the one round TC RNA amplification method generated significantly higher total hybridization signal intensity for each clone as compared to the aRNA method. The one round aRNA protocol did not generate significant hybridization signal intensity with either purification method. In conjunction with the TC RNA amplification without second strand synthesis method, the drop dialysis procedure produced higher average hybridization signal intensity (ranging from 1 × 10^8^ – 3.5 × 10^8^) compared to the column filtration method (5 × 10^7^ – 8 × 10^7^). Interestingly, hybridization signal intensity increased with decreasing amounts of input RNA for the drop dialysis method (Figure 5A), suggesting that this procedure works best when employing minute input amounts of RNA. In contrast, the column filtration method did not have this same trend at the four RNA concentrations tested (Figure 5B). Greater variability in hybridization signal intensity was observed using drop dialysis in conjunction with the TC RNA amplification and aRNA methods. For the aRNA amplification method, drop dialysis hybridization signal intensities ranged from 1 × 10^6^ – 2 × 10^7^ as compared to a 2.6 × 10^6^ – 7.6 × 10^6^ range for the column filtration method (Figure 5A, B). Additionally, the drop dialysis method displayed higher negative control hybridization signal intensity levels in comparison to the column filtration method for both the aRNA and TC amplification methods. Specifically, when hybridization signal intensity above negative controls values were compared between the purification methods, column filtration gave a consistent and increased percentage of clones displaying higher total spot signal intensity at the 4 concentrations than the drop dialysis method (Figure 5C, D). Furthermore, the TC RNA amplification method displayed statistically significantly higher total spot signal intensity above negative control background as compared to the aRNA procedure at all input RNA levels, whereas with the aRNA procedure extensive signal loss was observed on most lower-expressed clones when hybridization signal intensity was subtracted from negative control values (Figure 5C, D).

## 3. Discussion

In the present report, we have assessed the performance of two RNA amplification methods, TC RNA amplification and the aRNA procedure, at several low nanogram RNA concentrations that mimic levels obtained via laser capture microdissection (LCM) and related tissue microdissection technologies. Both of these RNA amplification methods use a linear amplification procedure based on generating a cDNA hybrid with a bacteriophage transcription promoter, which is subsequently used for IVT. However, the aRNA amplification method utilizes a T7 promoter linked to a poly d(T) primer, whereas the TC RNA amplification procedure has the ability to generate sense and antisense amplified RNA by enabling the T7 promoter sequence to be attached to either the 5’ or 3’ primers used in the amplification scheme [6].

TC RNA amplification and aRNA have been used in combination with microaspiration and/or LCM and subsequent microarray analysis by several independent research groups to examine gene expression levels within specific brain regions and cell types, including hippocampal and basal forebrain cholinergic neurons at the single cell level on a variety of microarray platforms ranging from moderate density custom-designed arrays to commercial high density oligonucleotide arrays [19, 22–29]. Notably, RNA amplification procedures have also been used extensively in cancer biology in addition to varied and diverse areas of biomedical research [30–34], indicating that these methods have broad appeal for multiple disciplines and research endeavors. The aRNA method (typically utilizing two rounds of amplification) has been employed for many microarray-based studies, with typical input RNA amounts ranging from a lower limit of 50 ng upwards to above 200 ng into the microgram range [31, 33, 35]. This indicates that sensitivity can be an issue, depending on the array platform and hybridization conditions utilized. The RNA input requirement has made it difficult to examine some types of cells via aRNA, especially those cell types that are rare and/or difficult to identify [32]. Evaluating single cells and/or rare populations of phenotypically-identified neurons for downstream genetic analyses is highly desirable. For this reason, we chose to compare the performance of aRNA and TC RNA amplification at relatively low starting input RNA concentrations, rather than concentrate on high levels of input RNA where virtually any RNA amplification scheme will perform adequately (assuming an RNA amplification method is necessary and/or required by the investigator). Results indicated no deterioration in signal quality when examining as little as 1 ng of starting RNA with the TC protocol. However, the aRNA protocol displayed a statistically significant lower signal to noise ratio when compared with the TC protocol all levels of starting RNA concentrations, demonstrating that for small amounts of starting RNA, the TC RNA amplification protocol yields a more robust signal on an array platform. Moreover, the hybridization signal intensity of the TC RNA amplification methods is similar to results published previously by our group using a second strand synthesis step [6].

Two types of cDNA purification methods were also investigated to determine whether either was more advantageous within the amplification schemes. Utilizing column filtration did not produce the highest raw hybridization signal intensity values. However, when integrating array signal with corresponding negative control background hybridization, a stringent method that the authors urge strongly to be used universally [4], this method tended to outperform the drop dialysis scheme in terms of positive identification of individual genes. Moreover, there are several different types of commercially available columns that can filter cDNAs and proteins at varying size limitations. This ability to impart size exclusion limitations upon cDNAs during an RNA amplification procedure may prove to be quite useful for reducing the amounts of unbound primers, and ultimately background nonspecific hybridization, without loss of the desired cDNA product.

## 4. Materials and Methods

### 4.1. Tissue and RNA accession

The animal protocols have been approved by the Institutional Animal Care and Use Committee (IACUC) of the Nathan Kline Institute/NYU School of Medicine and were in full accordance with NIH guidelines. Wild-type C57BL/6 mice (6–10 months old) were euthanized by cervical dislocation and the brain was removed, frozen on dry ice, and stored at –80 °C. Approximately 50–100 mg of cortical tissue was utilized for RNA extraction. Trizol (Invitrogen, Carlsbad, CA) was added at 10X (w/v) and tissue was homogenized manually. RNA was then extracted with chloroform and precipitated utilizing isopropanol and resuspended in 18.2 mega Ohm RNase-free water (Nanopure Diamond, Dubuque, IA). RNA purity and concentration was analyzed utilizing the total RNA Nano procedure with the Agilent 2100 bioanalyzer (Agilent Technologies, Palo Alto, CA).

### 4.2. Amplification of RNA utilizing TC RNA amplification procedure

The TC RNA amplification methodology was developed in this laboratory [6]. Presently, the protocol has been modified to obviate the need for second-strand cDNA synthesis [20]. RNAs (1, 10, 25, or 50 ng) were reverse transcribed in a solution containing poly d(T) primer (100 ng/μL) and TC primer (100 ng/μL) in 1X first strand buffer (Invitrogen), 2 ug of linear acrylamide (Applied Biosystems, Foster City, CA), 10 mM dNTPs, 100 μM DTT, 20 U of SuperRNase Inhibitor (Applied Biosystems), and 200 U of reverse transcriptase (Superscript III, Invitrogen). Single-stranded cDNAs were then subjected to RNase H digestion and re-annealing of the primers to generate cDNAs with double-stranded regions at the primer interfaces. Single stranded cDNAs were digested by adding the following and then placed in a thermal cycler: 10 mM Tris (pH 8.3), 50 mM KCl, 1.5 mM MgCl_2_, and 10 U RNase H (Invitrogen) in a final volume of 100 μL. RNase H digestion step at 37 °C, 30 minutes; denaturation step 95 °C, 3 minutes; primer re-annealing step 60 °C, 5 minutes. Samples were purified either by column filtration (Montage PCR filters; Millipore, Billerica, MA) or by drop dialysis. These purification methods were selected because they consist of a filter filtration system, rather than a conventional gel filtration. Specifically, employing a gel filtration matrix tends to give a low yield for cDNA purification, making RNA amplification quite difficult and hard to reproduce. For column filtration, column reservoirs were filled with 300 μL of 18.2 mega Ohm RNase-free water and the cDNA reaction was then added to the reservoir. The columns were then spun at 1000 × g for 15 minutes. To recover the cDNA, 20 μL of 18.2 mega Ohm RNase-free water was added to the columns, and the columns were inverted into clean microfuge tubes and spun at 1,000 × g for 2 minutes. The volume of the samples was measured and adjusted to 19 μL by speed vacuum and resuspension in 18.2 mega Ohm RNase-free water. For drop dialysis, phenol:chloroform extracted and ethanol precipitated cDNAs were resuspended and drop dialyzed on 25 um filter membranes (Millipore) against 50 ml 18.2 mega Ohm RNase-free water for 2 hours [36]. Samples were collected off the dialysis membrane and the volume was adjusted to 19 μL by speed vacuum and resuspension in 18.2 mega Ohm RNase-free water.

### 4.3. Amplification of RNA utilizing the aRNA amplification procedure

The aRNA amplification protocol utilized herein was modified from the Eberwine protocol and utilized by our group prior to the development of the TC RNA amplification procedure [2, 36]. Briefly, mRNAs were reverse transcribed in the presence of oligo d(T) (100 ng/μL) in 1X first strand buffer, 2 μg of linear acrylamide, 10 mM dNTPs, 100 μM DTT, 20 U of SuperRNase Inhibitor, and 200 U of reverse transcriptase (Superscript III). Second-strand cDNA was generated by adding the following to the first-strand cDNA reaction: 50mM Tris-HCl (pH 7.2), 10 mM MgSO_4_, 100 μM DTT, 15 mM dNTPs, 0.5 U T4 DNA ligase (Invitrogen), 10 U DNA polymerase I (Promega, Madison, WI), and 10 U RNase H. Samples were incubated at 16 °C for 2 hours, and completed by adding 10 U of T4 DNA polymerase (Promega) for 5 minutes. Reactions were stopped by the addition of 50 mM EDTA. Samples were purified by column filtration or drop dialysis as described for TC RNA amplification.

### 4.4. T7 amplification and hybridization to custom-designed cDNA array platforms

Hybridization probes were synthesized either from TC RNA amplification or aRNA products by IVT using ^33^P incorporation in 40 mM Tris (pH 7.5), 6 mM MgCl_2_, 10 mM NaCl, 2 mM spermidine, 10 mM DTT, 2.5 mM ATP, GTP and CTP, 100 uM of cold UTP, 20 U of SuperRNase Inhibitor, 2 KU of T7 RNA polymerase (Epicentre, Madison, WI), and 120 uCi of ^33^P -UTP (Perkin-Elmer, Boston, MA) [6, 36]. The reaction was performed at 37 °C for 4 hours. Radiolabeled TC RNA or aRNA probes were hybridized to custom-designed cDNA arrays without further purification.

### 4.5. Custom-designed cDNA array platforms and data analysis

Array platforms consisted of 1 μg of linearized cDNA purified from plasmid preparations adhered to high-density nitrocellulose (Hybond XL, GE Healthcare, Piscataway, NJ). Each cDNA and/or expressed sequence-tagged cDNA (EST) was verified by sequence analysis and restriction digestion. cDNA clones and ESTs from mouse, rat, and human were employed. Approximately 576 cDNAs/ESTs were utilized on the current array platform. Arrays were prehybridized (2 hours) and hybridized (14–16 hours) in a solution consisting of 6X SSPE, 5X Denhardt's solution, 50% formamide, 0.1% sodium dodecyl sulfate (SDS), and denatured salmon sperm DNA (200 μg/mL) at 42 °C in a rotisserie oven [4]. Following hybridization, arrays were washed sequentially in 2X SSC/0.1% SDS, 1X SSC/0.1% SDS and 0.5X SSC/0.1% SDS for 15 minutes each at 37 °C. Arrays were placed in a phosphor screen for 24 hours and developed on a phosphor imager utilizing the same settings for each array (GE Healthcare). Hybridization signal intensity was determined by utilizing ImageQuant TL array analysis program (GE Healthcare). The program quantifies the signal intensity, subtracts the background by utilizing the spot edge average for each clone and normalizes the hybridization signal intensity. The arrays for either the TC RNA amplification or the aRNA protocols were then compared to negative control arrays performed utilizing the respective protocols without any input RNA [4]. Hybridization signal intensity for these negative control samples were then subtracted from the arrays for each of the RNA concentrations. The statistical procedures for custom-designed microarray analysis have been described in detail elsewhere [4, 6]. Multiple (3–5) arrays were assayed for each purification method at each input RNA concentration (1, 10, 25 and 50 ng). Briefly, expression of TC amplified RNA bound to each linearized cDNA (approximately 576 cDNAs/ESTs) was expressed as a ratio of the total hybridization signal intensity of the array (a global normalization approach). Global normalization effectively minimizes variation due to differences in the specific activity of the synthesized TC/aRNA probe as well as the absolute quantity of probe present. Data analyzed in this manner does not allow the absolute quantitation of mRNA levels. However, an expression profile of relative changes in mRNA levels was generated. Relative changes in total hybridization signal intensity and percentage of cDNA clones above negative control were analyzed by one-way analysis of variance (ANOVA) with post-hoc analysis (Neumann-Keuls test; level of significance was set at p < 0.05) and correction for multiple observations for individual comparisons.

### 4.6. Real-time quantitative PCR (qPCR)

qPCR was performed in triplicate on the same microdissected frozen tissue samples subjected to the RNA amplification procedures. Samples were assayed on a real-time PCR cycler (7900HT, Applied Biosystems). Mouse TaqMan hydrolysis probes designed against the AMPA glutamate receptor subunit 1 (GRIA1) (Mn00514377_m1), beta actin (ACTB; Mm00447557_m1), and glyceraldehyde-3 phosphate dehydrogenase (GAPDH; Hs00266705_g1) were utilized (Applied Biosystems). Standard curves and cycle threshold (Ct) were generated using standards obtained from total mouse brain RNA. The ddCT method was employed to determine relative gene level differences. A total of 3–4 independent samples per RNA concentration were assayed in triplicate. Negative controls were used for each assay, and consisted of the reaction mixture without input RNA.

## 5. Conclusions

As we move forward with the use of high-throughput functional genomics approaches in biomedical sciences, the ability to manipulate minute starting amounts of RNA has become a critical parameter. The TC RNA amplification method employed herein can be utilized not only for examining different cell types within brain, but for assessing differential gene expression within virtually any cell type utilizing any tissue or cell culture paradigm under normative and pathological conditions. The lack of a requirement for second strand synthesis in the TC RNA amplification procedure is significant, as fewer steps in the procedure is economical, and a decreased likelihood of yield loss is desirable, especially for small and/or precious samples.
